# Investigation of Intraoperative and Permanent Diagnostic Consistency in Glial Tumors Considering Rater and Technical Variability

**DOI:** 10.3390/medicina61091592

**Published:** 2025-09-03

**Authors:** Mine Ozsen, Ilker Ercan, Selva Kabul, Rabia Dolek

**Affiliations:** 1 Department of Pathology, Faculty of Medicine, Bursa Uludag University, Bursa 16059, Türkiye; m.isikoglu@hotmail.com (M.O.); selva@uludag.edu.tr (S.K.); 2 Department of Biostatistics, Institute of Health Sciences, Bursa Uludag University, Bursa 16059, Türkiye; 3 Department of Biostatistics, Faculty of Medicine, Bursa Uludag University, Bursa 16059, Türkiye; 4 Department of Pathology, Faculty of Medicine, İstinye University, İstanbul 34010, Türkiye; dolekrabia@gmail.com

**Keywords:** frozen, generalizability theory, glial tumors, intraoperative consultation, reliability

## Abstract

*Background and Objectives:* One of the most critical areas of measurement and evaluation in healthcare is pathological evaluation, especially intraoperative consultation. Studies conducted to identify sources of error in this field are usually one-sided; however, in evaluation processes with multiple sources of error, such as intraoperative consultation, generalizability theory can evaluate these sources of error simultaneously in a single analysis, thereby contributing to the field. In this study, the reliability of intraoperative and permanent histopathological evaluations of glial tumors was analyzed using generalizability theory to identify the sources of error in the observed evaluation inconsistencies. *Materials and Methods:* The study included 319 glial tumor cases that underwent intraoperative evaluation and were analyzed using generalizability theory. Three pathologists performed independent evaluations in two stages. *Results:* The reliability coefficient calculated for all cases was 0.9234 without radiological information and 0.9243 after learning the radiological information. The reliability coefficient was 0.8875 and 0.8989, respectively, in cases over 18 years of age, and 0.8845 and 0.9062 in cases under 18 years of age. These findings indicate that the addition of radiological information to the evaluation resulted in a slight increase in reliability, particularly in cases under 18 years of age. In all of our reliability assessments for different conditions, the highest variability was found to originate from the rater. *Conclusions:* The findings suggest that intraoperative evaluation demonstrates a high degree of reliability in the pathological assessment of glial tumors. When differences between the rater and the technique are evaluated together, it is observed that the rater has a more significant impact on reliability. While radiological information is generally considered a factor that increases reliability, it is partially more effective, especially in cases involving individuals under the age of 18, which highlights the importance of multidisciplinary data sharing in intraoperative diagnostic processes.

## 1. Introduction

The possibility of error in all measurement processes is a known and always important consideration. Errors in health research are particularly significant because they can impact diagnostic and/or treatment performance, potentially leading to undesirable outcomes for patients [1].

One of the most critical areas of measurement and evaluation in healthcare is pathological assessment. In this context, intraoperative consultation (frozen section) holds particular importance. Despite advances in neuroimaging techniques for central nervous system lesions, the need for intraoperative consultation persists. Frozen assessment is widely used in clinical practice for evaluating central nervous system lesions in various aspects, including guiding the surgeon, planning surgical treatment, determining whether the sampled area represents the target lesion, and assessing the adequacy of the sample [2,3,4].

Despite the advantages offered by the intraoperative consultation technique, it also has some limitations. These include the heterogeneity of lesions, errors made by the surgeon, pathologist, or technician, and technical problems (cautery, crushing, freezing, or drying artifacts). Both the limitations of the intraoperative consultation technique and the presence of lesions within the central nervous system, which require particular experience and knowledge and can create diagnostic difficulties, cause inconsistencies between the intraoperative diagnosis and the permanent diagnosis based on neuropathology materials. Different studies in the literature have reported frozen diagnostic accuracy rates ranging from 78.4% to 95% [5,6]. This wide range of accuracy rates indicates a need for more studies on the subject. When evaluating accuracy, it is important to perform comprehensive analyses that take into account factors such as laboratory conditions and rater effect.

In evaluation processes with multiple sources of error, such as intraoperative consultation, generalizability theory (G Theory) stands out as it offers the possibility of statistically analyzing these sources of error. G theory enables the identification of causes for inconsistencies in measurement results. With a single analysis, different error sources and their interactions can be evaluated. Thus, more comprehensive information about the reliability of the measurement process can be obtained, and a reference can be created for efforts to reduce errors [7,8,9]. In the medical literature, applications of G-theory have primarily focused on educational assessment, technical skill evaluation, and patient–physician communication, whereas its use in direct clinical practice has remained limited [10,11]. Preuss et al. applied G-theory to develop reliable clinical assessment protocols and showed that it outperformed Classical Test Theory by enabling the simultaneous evaluation of multiple sources of error, the generalization across different measurement conditions, and the recalculation of reliability [12]. To the best of our knowledge, no studies to date have specifically applied G-theory in the field of pathology.

Previous studies examining the diagnostic accuracy of intraoperative consultation in central nervous system lesions have generally reported accuracy rates ranging from 78% to 95%. However, these studies have significant limitations, because they often investigate sources of error in a one-dimensional manner, focusing solely on differences between evaluators or on technical issues. Such approaches may fail to comprehensively account for the numerous and interrelated sources of variability that arise simultaneously in clinical practice. Generalizability theory (G-theory) offers a methodological advantage in this regard, as it enables the analysis of multiple sources of error—such as case, evaluator, and technical factors—and their interactions within a single framework. In this study, we aimed to evaluate the overall reliability coefficients for intraoperative and permanent diagnoses of glial tumors using G-theory, determine the effect of incorporating radiological information into the evaluation process, and investigate whether reliability differs between pediatric and adult cases.

## 2. Materials and Methods

### 2.1. Study Group

Between January 2010 and February 2024, reports in the archives of the pathology department were reviewed via the electronic hospital database, and cases of glial tumors that underwent intraoperative frozen section evaluation were identified. Specimens from the included cases were retrieved from the pathology archive, and their eligibility for re-evaluation was assessed. The necessary clinical data (age, gender, radiological findings, localization) of the cases were obtained from electronic files, and pathological data (histopathological diagnosis and localization) were obtained from pathology reports. All cases diagnosed with glial tumors and undergoing intraoperative frozen section evaluation were included in the study, regardless of age and gender, while cases diagnosed with glial tumors without intraoperative frozen section evaluation, cases diagnosed with conditions other than glial tumors, cases with unavailable clinical data or specimens of insufficient quality for re-evaluation, and cases with inaccessible specimens in the archive were excluded from the study. Based on these criteria, the study group was determined to consist of 319 cases.

### 2.2. Evaluation Process

Frozen sections from each case were independently evaluated by three different expert pathologists. The professional experience of the pathologists was 10, 7, and 2 years. The evaluation was performed in terms of both histopathological diagnosis and tumor grade. In the first stage, the evaluation was performed without radiological information. The same evaluation process was repeated in the second stage after the radiological findings were made known. There were three months between the two stages. During the evaluation process, the raters were kept independent of each other and the original diagnoses.

### 2.3. Statistical Analysis

The study was conducted using the generalizability theory experimental design. The experimental design had a cross-over structure (c × t × o) consisting of cases (c) with two different techniques (t: permanent sections and frozen sections) for evaluating tissue samples obtained from tissues, followed up by expert pathologists (r: three expert pathologists). The total variance components related to differences in assessment are summarized in Table 1.

To calculate the G coefficient, estimates of the variance components were obtained using the expected mean square rules with the c × t × o design. A G coefficient close to 1.0 indicates high reliability, with values above 0.80 generally considered acceptable for clinical decision-making [13,14].

$$\sigma_{\mathrm{crt},e}^{2}\;=\;{\mathrm{MS}}_{\mathrm{crt},e}$$

$$\sigma_{\mathrm{cr}}^{2}\;=\;({\mathrm{MS}}_{\mathrm{cr}}\;-\;{\mathrm{MS}}_{\mathrm{crt},e})/n_{t}$$

$$\sigma_{\mathrm{ct}}^{2}\;=\;({\mathrm{MS}}_{\mathrm{ct}}\;-\;{\mathrm{MS}}_{\mathrm{crt},e})/n_{r}$$

$$\sigma_{\mathrm{rt}}^{2}\;=\;({\mathrm{MS}}_{\mathrm{rt}}\;-\;{\mathrm{MS}}_{\mathrm{crt},e})/n_{p}$$

The variance components given above are sufficient for calculating the G coefficient. However, in a two-way crossover design model, additional variance components must be calculated. These components are estimated as follows:

$$\sigma_{c}^{2}\;=\;({\mathrm{MS}}_{c}\;-\;{\mathrm{MS}}_{\mathrm{cr}}\;-\;{\mathrm{MS}}_{\mathrm{ct}}\;+\;{\mathrm{MS}}_{\mathrm{crt},e})/n_{r}n_{t}$$

$$\sigma_{r}^{2}\;=\;({\mathrm{MS}}_{r}\;-\;{\mathrm{MS}}_{\mathrm{cr}}\;-\;{\mathrm{MS}}_{\mathrm{rt}}\;+\;{\mathrm{MS}}_{\mathrm{crt},e})/n_{c}n_{t}$$

$$\sigma_{t}^{2}\;=\;({\mathrm{MS}}_{t}\;-\;{\mathrm{MS}}_{\mathrm{ct}}\;-\;{\mathrm{MS}}_{\mathrm{rt}}\;+\;{\mathrm{MS}}_{\mathrm{crt},e})/n_{c}n_{r}$$

Relative and absolute error variances are estimated as follows:

σ^Rel2 = (σcr2/nr) + (σct2/no) + (σct,e2/nrnt)

σ^Abs2 = (σr2/nr) + (σt2/nt) + (σrt2/nrnt) + σRel2

The G coefficients are estimated as follows:

G = σp2/(σp2 + σ^Rel2)

The normality of the data distribution was examined using the Shapiro–Wilk test. Due to the non-normal distribution, the descriptive statistics are given as “median (min:max)” (*p* < 0.05). Descriptive statistics for categorical data are presented as numbers (n) and percentages (%). All statistical analyses were performed using IBM SPSS Statistics version 29.0.

### 2.4. Ethical Approval

Approval for the study was obtained from the local ethics committee (decision dated 20 March 2024, numbered 2024-4/10) and the study was conducted in accordance with the Helsinki Declaration.

## 3. Results

The median age of the 319 cases included in the study was 39. The minimum age was 1, while the maximum age was 89. When the cases were grouped into pediatric and adult groups based on age (under 18 and over 18), 90 cases (28.2%) were under 18, while 229 cases (71.8%) were over 18. Of the cases, 142 (44.5%) were female and 177 (55.5%) were male. The female-to-male ratio was 0.8.

When the cases were evaluated in terms of tumor localization, 253 (79.8%) tumors were supratentorial, 58 (18.3%) tumors were infratentorial, and 6 (1.9%) tumors were spinal. Demographic and clinical data for the cases are summarized in Table 2.

All cases with and without radiological information are summarized in Table 3 and Table 4. The highest variance component was found to be related to the case (c) variable with a rate of 81.55%. The variance between pathologists (r) was 0.02%, while the variance due to technique (t) was quite low at 0.01%. The pathologist–case interaction (r × c) contributed 11.82% to the variance, the case–technique interaction (c × t) contributed 3.2%, and the pathologist–technique interaction contributed 0.17% to the variance. When radiological information was included in the evaluation, the case-related variance increased to 84.69%, while the other variance components remained similar. The reliability coefficient was similar without radiological information (G = 0.9234) and after learning radiological information (G = 0.9243).

The results obtained from the evaluation based on cases over the age of 18 are presented in Table 5 and Table 6. According to these results, the case variance was the highest variance component with 78.31% in the analysis that was made without radiological information; the c × r interaction was calculated as 13.46%; the c × t interaction as 4.05%; and the r × t interaction as 0.25%. With the addition of radiological information, case variance increased to 80.71%, while c × r interaction decreased to 11.86%. The reliability coefficient showed a slight difference when radiological information was included (G = 0.8875 without radiological information, G = 0.8989 after radiological information was learned).

The results obtained from the evaluation based on cases under the age of 18 are presented in Table 7 and Table 8. According to these results, the case variance was determined as 77.21% in the analysis that was made without radiological information; the c × r interaction was 14.86%; the c × t interaction was 3.4%; and the r × c interaction was 0.001%. After adding radiological information, the case variance increased to 81.58%, the c × r interaction decreased to 14.06%, and the error component decreased to 2.15%. The generalizability coefficient was 0.8845 without radiological information and 0.9062 after learning radiological information.

## 4. Discussion

Intraoperative consultation (frozen assessment) plays a critical role in managing and guiding the surgical process in routine neurosurgical practice. In this study, the reliability of frozen diagnosis was analyzed using generalizability theory, and the effects of factors that could influence reliability, such as rater and technical changes that could affect the process, were examined to assess the reliability levels of the diagnosis. The reliability coefficient calculated by considering all cases was 0.9234 without radiological information and 0.9243 after learning the radiological information. The reliability coefficient was 0.8875 and 0.8989, respectively, in cases over 18 years of age, and 0.8845 and 0.9062 in cases under 18 years of age. These findings reveal that the reliability level increased slightly, especially in cases under 18 years of age, when radiological information was added to the evaluation. In all of our reliability assessments for different situations, it was determined that the highest variability originated from the rater.

The histomorphological evaluation of glial tumors is a challenging area of pathology that requires experience. Various studies have shown differences between evaluators in terms of histopathological diagnosis and grade. In a study by Scott et al., the inter-evaluator agreement rate for glioblastoma was found to be 96%, while in a study by Aldape et al., it was reported that the disagreement rate between evaluators was 23% and that 16% of these disagreements resulted in significant clinical differences in patient management [15,16].

Although histopathological evaluation remains the gold standard for tumor diagnosis, it is not always sufficient on its own, especially in central nervous system lesions. Studies have shown that histopathological evaluation is more useful and accurate when combined with clinicoradiological data [17]. Rathore et al. evaluated the integration of data obtained from magnetic resonance imaging and histopathological evaluation in predicting overall survival in gliomas and observed that integration improved process prediction [18].

The study contributes to the field with its methodological originality. In the literature, interobserver agreement in the histopathological evaluation of glial tumors has generally been assessed using univariate analyses dependent on the raters. No analysis has been conducted using an approach that allows for simultaneous analysis of multiple error sources within a single analysis. Therefore, the simultaneous evaluation of multiple factors makes our study unique.

This study has some limitations. Firstly, it was conducted at a single center, which may restrict the generalizability of the findings to other institutions with different patient populations and laboratory conditions. Secondly, although three expert pathologists participated, the number of evaluators was limited, and inter-evaluator variability may differ with a larger or more diverse group. Future studies should aim to validate these findings in multicenter settings with larger evaluator groups. In addition, prospective designs and the integration of artificial intelligence-based image analysis could further strengthen the methodology.

## 5. Conclusions

Intraoperative (frozen) evaluation can be said to have a high level of reliability for the evaluation of glial tumors. When differences arising from the rater and technique are evaluated together, the rater is seen to have a more effective impact on reliability. While radiological information was found to be a factor that generally increases reliability, it was determined to be more effective in cases under the age of 18, emphasizing the importance of multidisciplinary data-sharing in intraoperative diagnostic processes.

## Figures and Tables

**Table 1:** The total variance components.

Symbols of Variance Components	Definitions of Variance Components
$\sigma_{c}^{2}$	Case-dependent variance (case by case variability)
$\sigma_{t}^{2}$	Technique-dependent variance (technique by technique variability)
$\sigma_{r}^{2}$	Rater-dependent variance (rater by rater variability)
$\sigma_{\mathrm{tr}}^{2}$	Technique-rater interaction variance
$\sigma_{\mathrm{tc}}^{2}$	Technical-case interaction variance
$\sigma_{\mathrm{rc}}^{2}$	Rater-case interaction variance
$\sigma_{\mathrm{cto},e}^{2}$	Technique-rater-case interaction variance and other error sources (not included in the experimental design)

**Table 2:** Demographic and clinical characteristics of the study population.

Variable	n (%)/Value
Total cases	319 (100%)
Pediatric (<18)	90 (28.2%)
Adult (≥18)	229 (71.8%)
Age (years)	39 (Min-max: 1–89)
Pediatric (<18)	10 (Min-max: 1–18)
Adult (≥18)	47 (Min-max: 19–89)
Sex	
Female	142 (44.5%)
Male	177 (55.5%)
Tumor localization	
Supratentorial	253 (79.8%)
Infratentorial	58 (18.3%)
Spinal	6 (1.9%)

**Table 3:** Variance components and reliability coefficients for all cases without radiological information.

Source of Variation	Degrees of Freedom	Mean of Squares	Estimated Variance Component	Percentage of Total Variance (%)	Reliability (G Coefficient)
Case (c)	318	4.777	1519.196	81.55	0.9234
Rater (r)	2	0.229	0.459	0.02	
Technique (t)	1	0.134	0.134	0.01	
Case × Rater (c × r)	636	0.346	220.208	11.82	
Case × Technique (c × t)	318	0.187	59.533	3.2	
Rater × Technique (r × t)	2	1.540	3.079	0.17	
Case × Rater × Technique, error (c × r × t, e)	636	0.095	60.254	3.23	

**Table 4:** Variance components and reliability coefficient in all cases with radiological information available.

Source of Variation	Degrees of Freedom	Mean of Squares	Estimated Variance Component	Percentage of Total Variance (%)	Reliability (G Coefficient)
Case (c)	318	5.203	1654.499	84.69	0.9243
Rater (r)	2	0.735	1.470	0.08	
Technique (t)	1	0.189	0.189	0.01	
Case × Rater (c × r)	636	0.313	199.196	10.19	
Case × Technique (c × t)	318	0.156	49.645	2.54	
Rater × Technique (r × t)	2	0.508	1.017	0.05	
Case × Rater × Technique, error (c × r × t, e)	636	0.075	47.650	2.44	

**Table 5:** Variance components and reliability coefficient in cases over 18 years of age without radiological information.

Source of Variation	Degrees of Freedom	Mean of Squares	Estimated Variance Component	Percentage of Total Variance (%)	Reliability (G Coefficient)
Case (c)	228	4.060	925.594	78.31	0.8875
Rater (r)	2	0.082	0.163	0.01	
Technique (t)	1	1.164	1.164	0.10	
Case × Rater (c × r)	456	0.349	159.170	13.46	
Case × Technique (c × t)	228	0.210	47.836	4.05	
Rater × Technique (r × t)	2	1.453	2.905	0.25	
Case × Rater × Technique, error (c × r × t, e)	456	0.099	45.095	3.82	

**Table 6:** Variance components and reliability coefficient based on radiological information in cases over 18 years of age.

Source of Variation	Degrees of Freedom	Mean of Squares	Estimated Variance Component	Percentage of Total Variance (%)	Reliability (G Coefficient)
Case (c)	228	4.316	984.086	80.71	0.8989
Rater (r)	2	0.547	1.093	0.09	
Technique (t)	1	1.107	1.107	0.09	
Case × Rater (c × r)	456	0.317	144.574	11.86	
Case × Technique (c × t)	228	0.208	47.393	3.89	
Rater × Technique (r × t)	2	0.539	1.079	0.09	
Case × Rater × Technique, error (c × r × t, e)	456	0.088	39.921	3.27	

**Table 7:** Variance components and reliability coefficient without radiological information in cases under 18 years of age.

Source of Variation	Degrees of Freedom	Mean of Squares	Estimated Variance Component	Percentage of Total Variance (%)	Reliability (G Coefficient)
Case (c)	89	3.338	297.067	77.21	0.8845
Rater (r)	2	0.739	1.478	0.38	
Technique (t)	1	1.252	1.252	0.33	
Case × Rater (c × r)	178	0.321	57.189	14.86	
Case × Technique (c × t)	89	0.147	13.081	3.4	
Rater × Technique (r × t)	2	0.002	0.004	0.001	
Case × Rater × Technique, error (c × r × t, e)	178	0.082	14.663	3.81	

The difference in total variance percentages is due to rounding.

**Table 8:** Variance components and reliability coefficient based on radiological information in cases under the age of 18.

Source of Variation	Degrees of Freedom	Mean of Squares	Estimated Variance Component	Percentage of Total Variance (%)	Reliability (G Coefficient)
Case (c)	89	3.682	327.659	81.58	0.9062
Rater (r)	2	0.763	1.526	0.38	
Technique (t)	1	0.363	0.363	0.09	
Case × Rater (c × r)	178	0.317	56.474	14.06	
Case × Technique (c × t)	89	0.078	6.970	1.73	
Rater × Technique (r × t)	2	0.007	0.015	0.003	
Case × Rater × Technique, error (c × r × t, e)	178	0.049	8.652	2.15	

The difference in total variance percentages is due to rounding.

## Data Availability

The data presented in this study are available on request from the corresponding author.
